# Supplementary material for: Self-cycled photo-Fenton-like system based on an artificial leaf with a solar-to-H2O2 conversion efficiency of 1.46%
Source: Nat Commun. 2022 Aug 25;13:4982. doi: 10.1038/s41467-022-32410-0 (PMC9411154; doi:10.1038/s41467-022-32410-0)
Supplement: Supplementary file 1 — Supplementary Information [file 41467_2022_32410_MOESM1_ESM.pdf]

Supplementary Information:

Self-cycled photo-Fenton-like system based on an artificial leaf with a solar energy conversion efficiency of 1.46%

C.R. Dong et al.

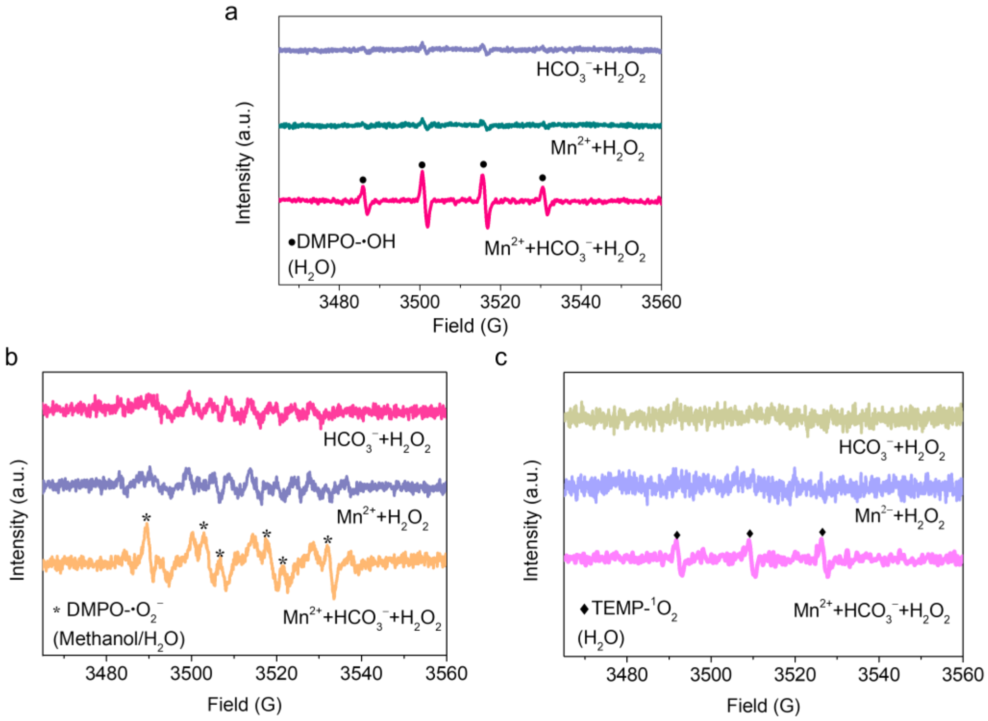

Supplementary Fig. 1. EPR response of (a) DMPO-·OH signal in H_2O electrolyte, (b) $\cdot O_2^-$ signal in methanol/H_2O electrolyte and (c) $^1O_2$ signal in H_2O electrolyte from the BAP system with/without Mn^{2+} or HCO_3^-. The interfere peak in (b) except for the $O_2^{\bullet -}$ is caused by the H_2O molecule in the aqueous solution (the appropriate electrolyte should be the methanol).

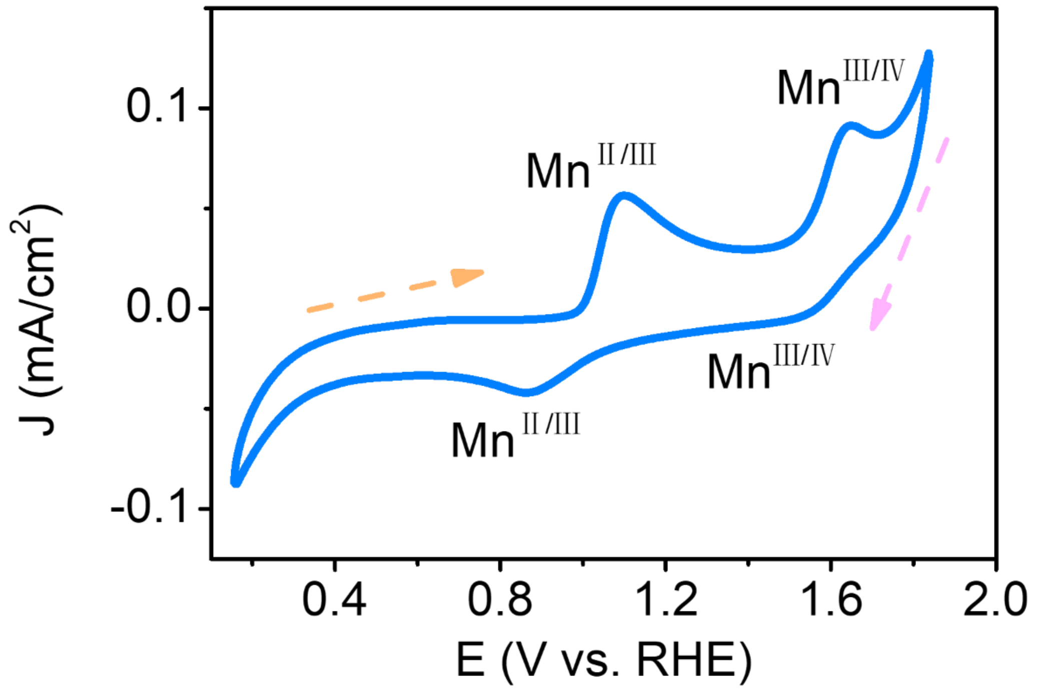

Supplementary Fig. 2. Cyclic voltammetry curve of 5 mM Mn(II) under 0.4M $NaHCO_3^-$ electrolyte.

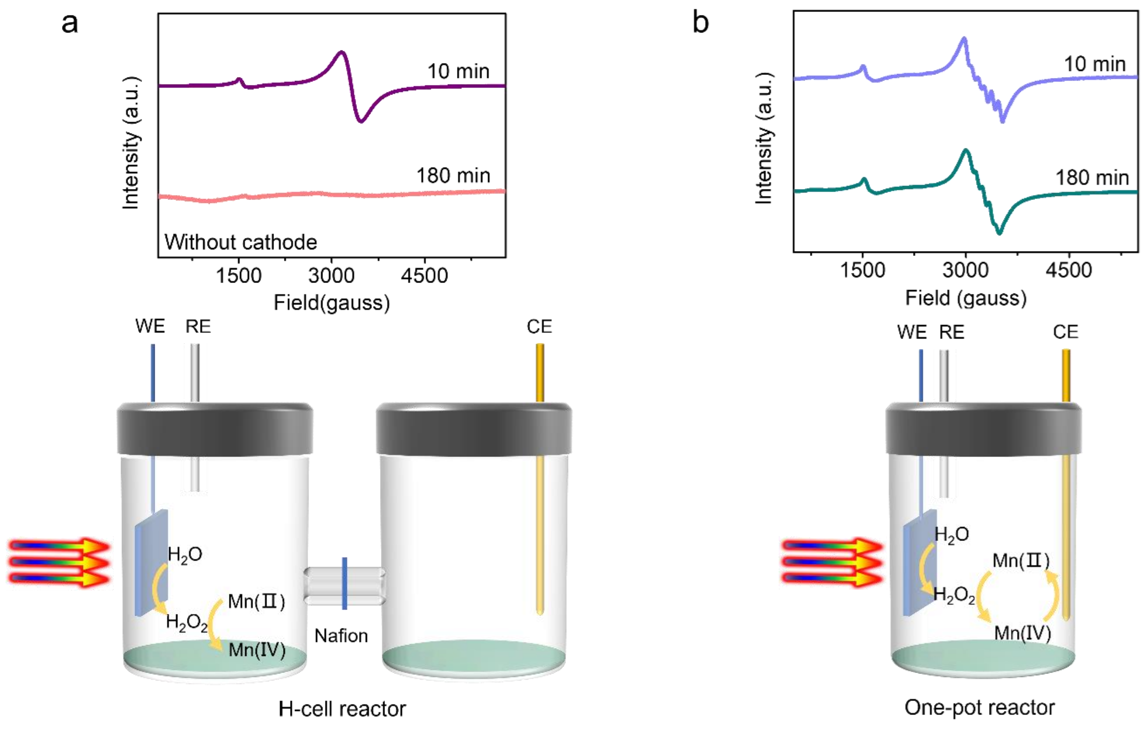

Supplementary Fig. 3. EPR response of Mn species under different time of a, without cathode in the H-cell reactor. b, with cathode in the one-pot reactor.

$$SHE = \frac{c(\mathrm{H_2O_2}) * \Delta G(\mathrm{H_2O_2})}{\mathrm{P}_{sun}}$$

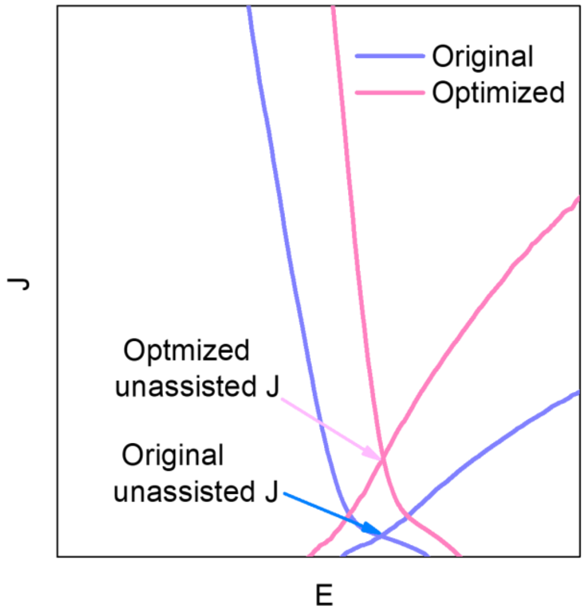

Supplementary Fig. 4. The theoretical operating point for a coupled photochemical reaction before and after optimization. Increasing the reaction kinetics (slope of the J-V curve), including cathodically shifting the onset potential of the photoanode, or anodically shifting the onset potential of the cathode would substantially raise the intersection point of the J-V curve of the photoanode and cathode, corresponding to a considerable increase in the working current of the coupled photochemical reaction under bias-free conditions. A higher operation current (J_{op}) corresponds to a higher H_2O_2 production rate ($c(H_2O_2)$) and solar-to-hydrogen-peroxide efficiency (SHyE).

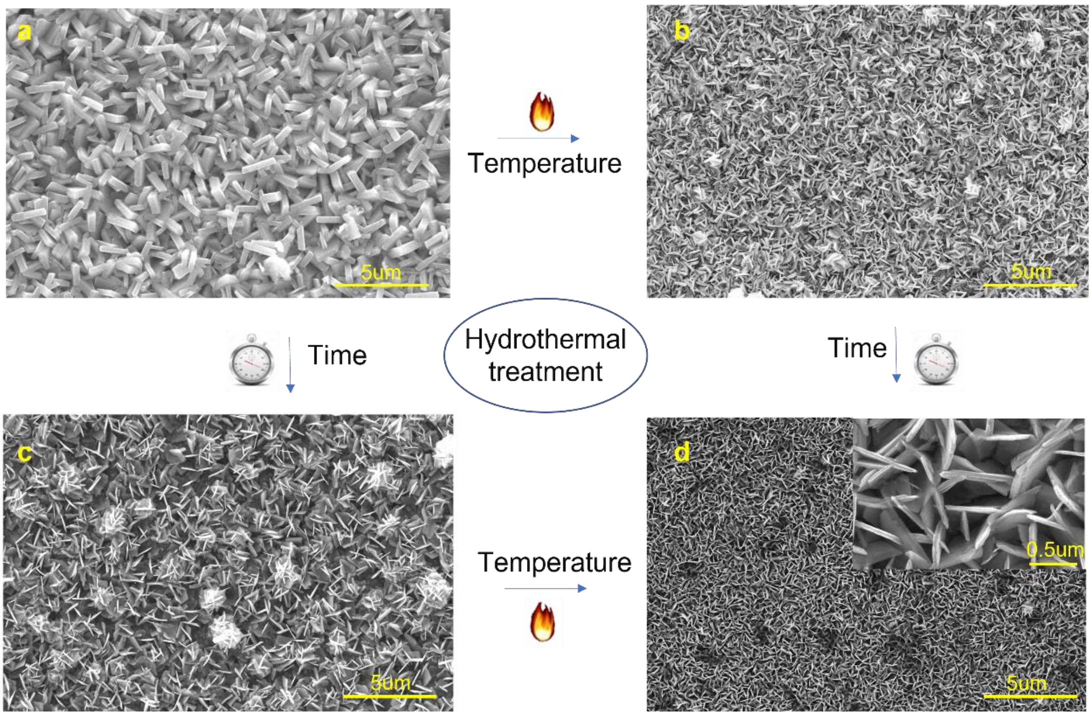

Sample	T (min)	Temperature (℃)
a	180	150
b	180	120
c	120	120
d	60	110

Supplementary Fig. 5. Texture engineering of WO_3 nanosheet arrays. Controlled experiments were conducted to tailor the morphology of WO_3 nanosheet arrays by varying the reaction temperature and time. Scale bar: 5 μm.

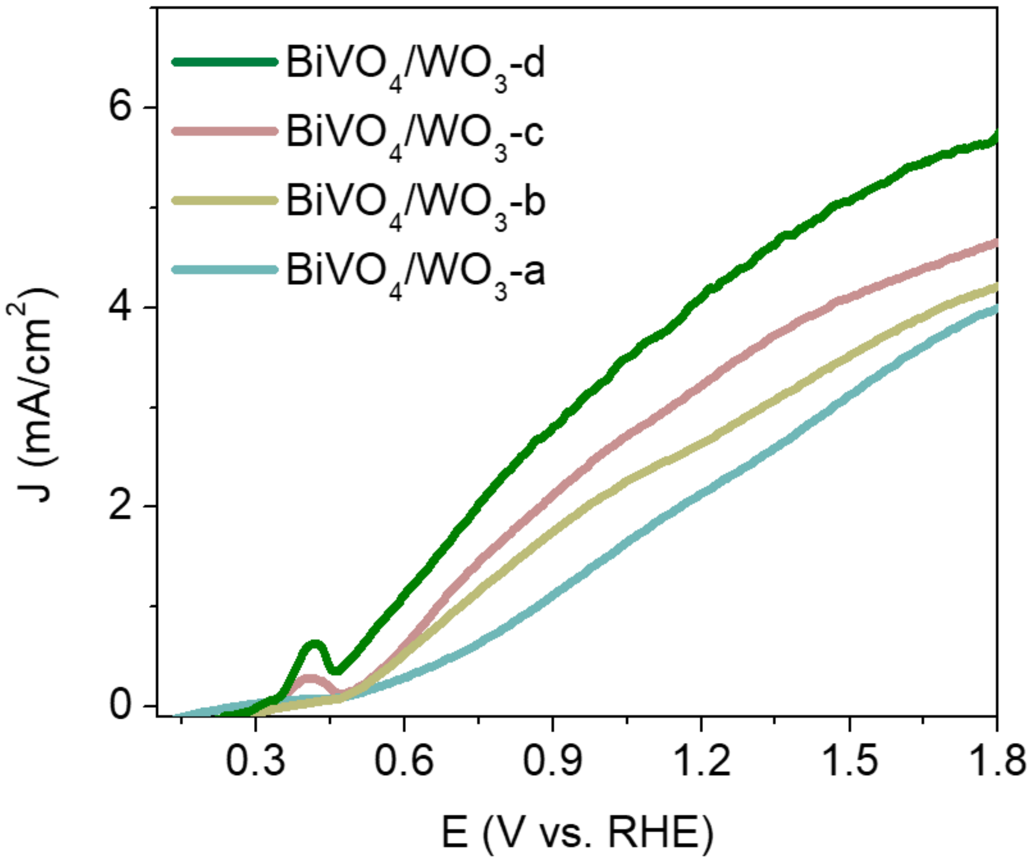

Supplementary Fig. 6. Electrochemical performance of the $BiVO_4/WO_3$ photoanode. LSV scans of the $BiVO_4/WO_3$ photoanode using different WO_3 substrates in Supplementary Fig. 5 under AM 1.5 illumination in 0.4 M $NaHCO_3$ electrolyte.

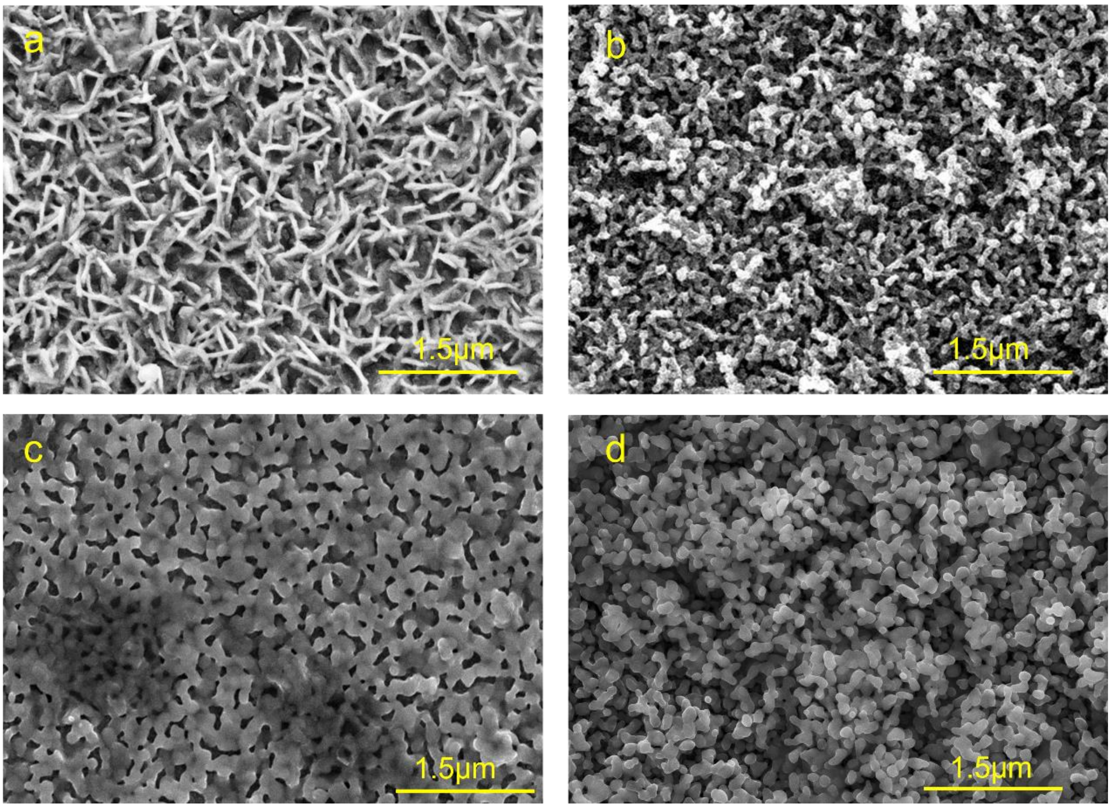

Supplementary Fig. 7. Synthesis of $BiVO_4/WO_3$. SEM image of $BiVO_4/WO_3$ photoanode with different precursor concentration of x=5, 10, 20 and 25 for a, b, c and d, respectively. Scale bar: 1.5 um.

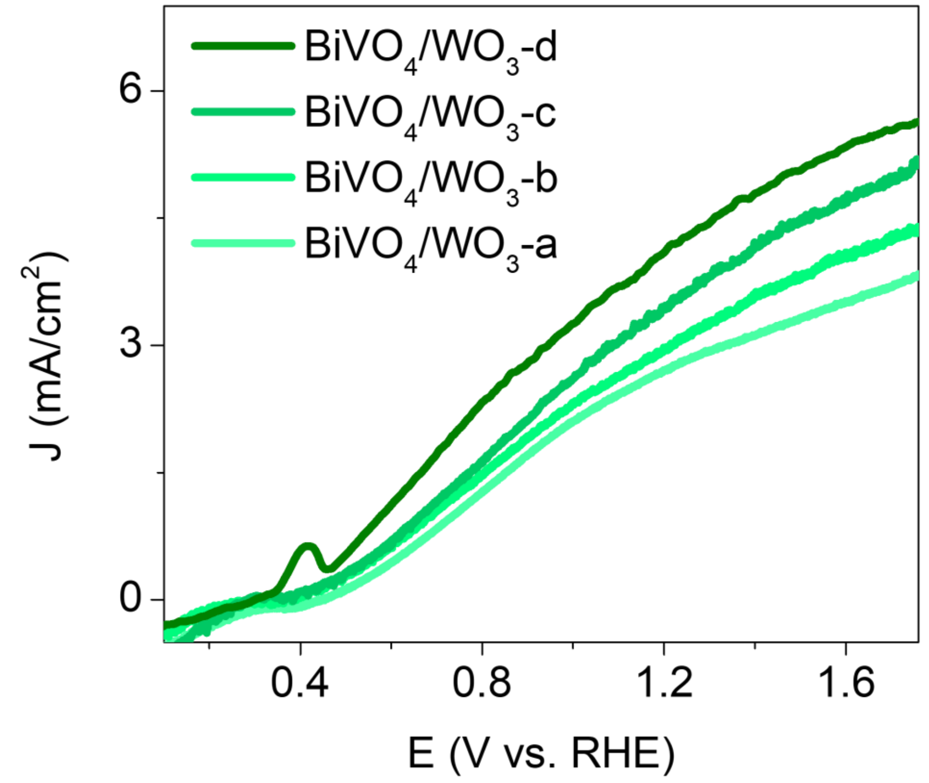

Supplementary Fig. 8. Electrochemical performance of the $BiVO_4/WO_3$ photoanode. LSV scans of the $BiVO_4/WO_3$ photoanode with various precursor concentrations of x=5, 10, 20 and 25, corresponding to a, b, c and d in Supplementary Fig. 7, respectively.

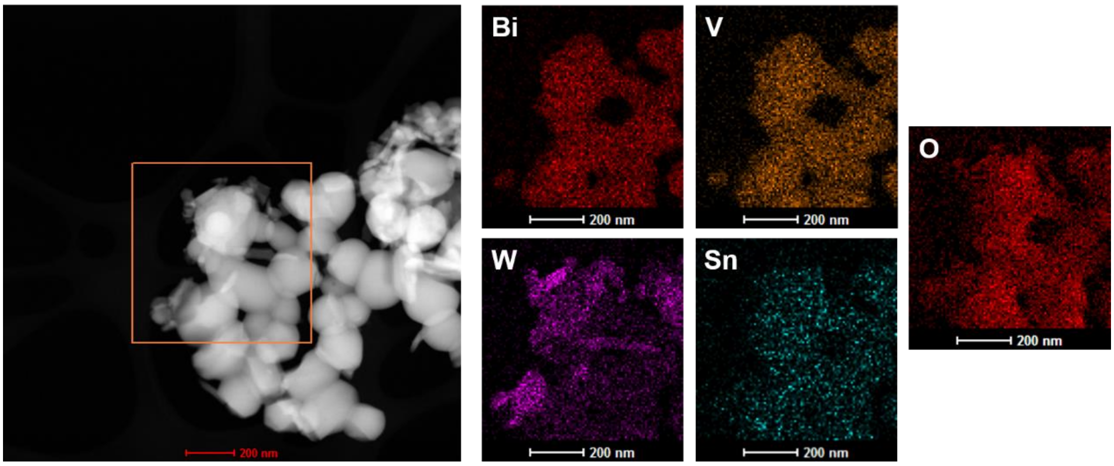

Supplementary Fig. 9. TEM EDS mapping of the $SnO_{2-x}/BiVO_4/WO_3$ photoanode. The porous mesoscopic structure of $SnO_{2-x}/BiVO_4/WO_3$ consisted of WO_3 nanosheets and $BiVO_4$ nanoparticles where the SnO_{2-x} layer was located.

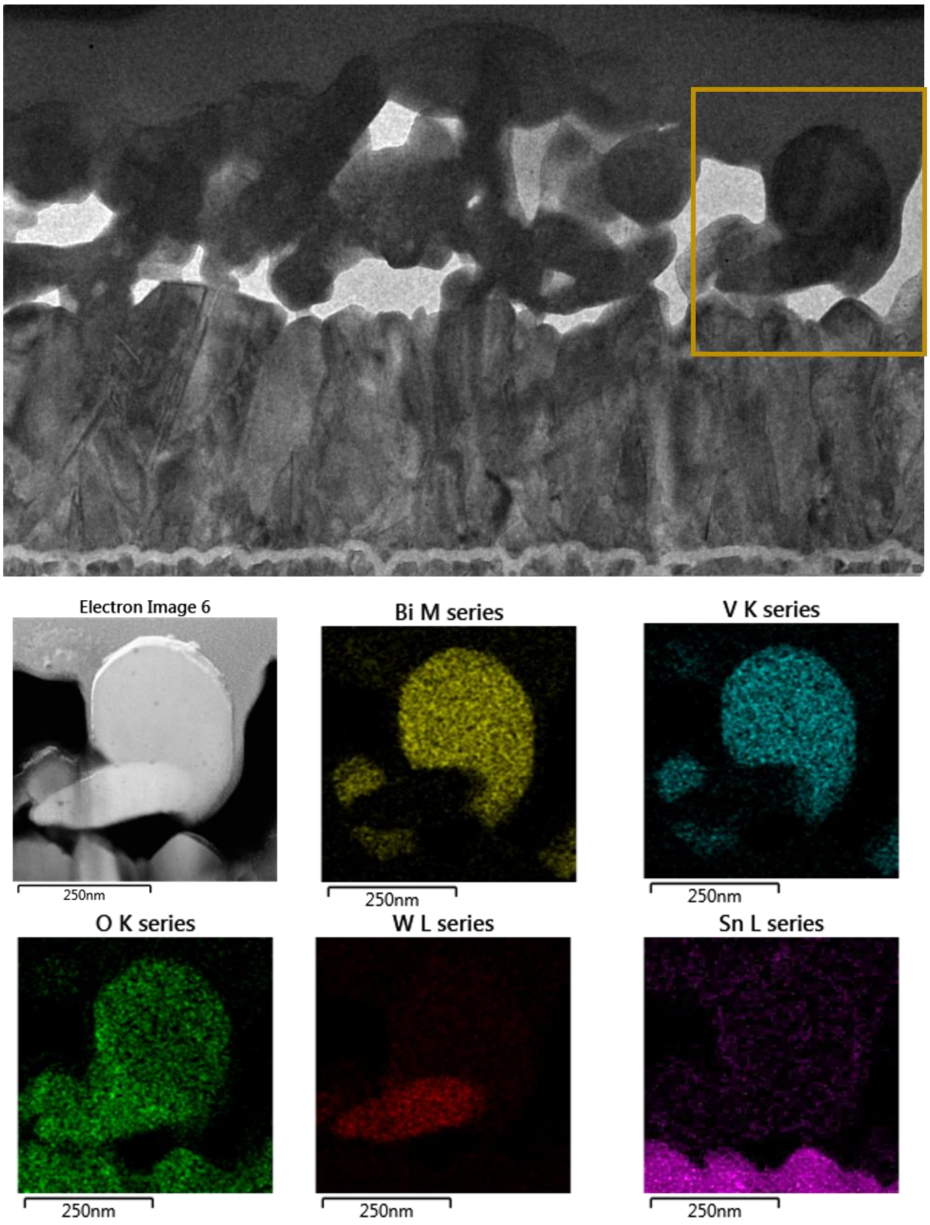

Supplementary Fig. 10. HAADF and STEM-EDS elemental mapping images showing cross-sectioned microstructures of the $SnO_{2-x}/BiVO_4/WO_3$ photoanode. Cross-sectional STEM-EDS of the $SnO_{2-x}/BiVO_4/WO_3$ photoanode *via* focused ion beam technology. It was observed Sn was mainly concentrated on the $BiVO_4$ particle surface, with clear distribution edges. However, due to the low mapping resolution from HAADF and very thin SnO_{2-x}, noise was inevitable.

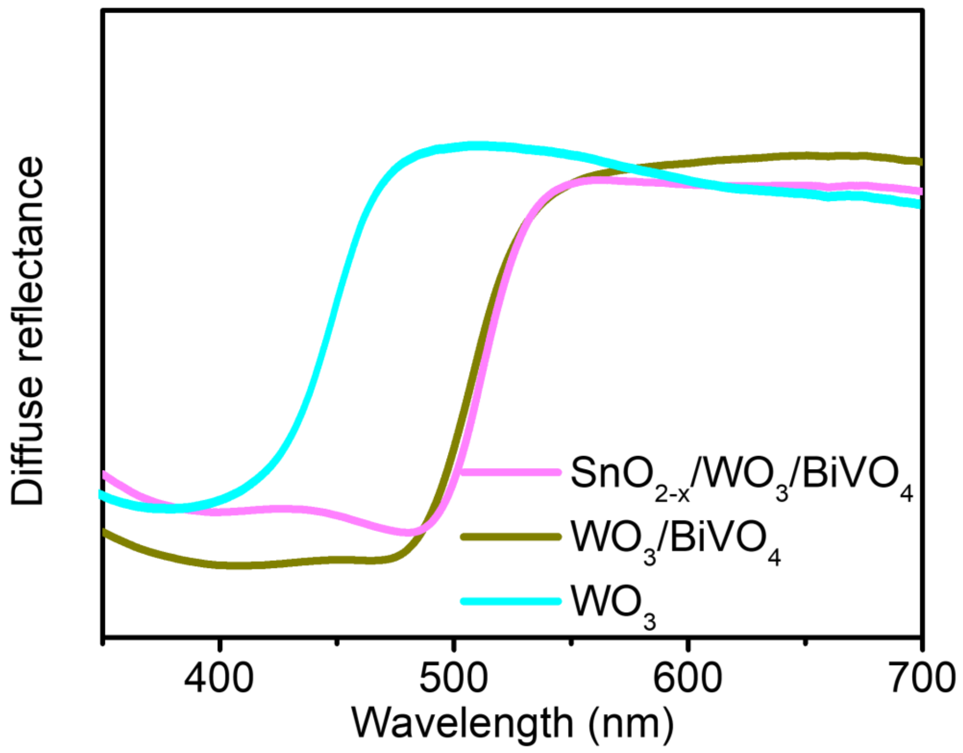

Supplementary Fig. 11. UV-vis absorption curves of WO_3, $BiVO_4/WO_3$ and $SnO_{2-x}/BiVO_4/WO_3$. $SnO_{2-x}/BiVO_4/WO_3$ has an absorption edge similar to that of $BiVO_4/WO_3$ but much greater than the absorption edge of WO_3.

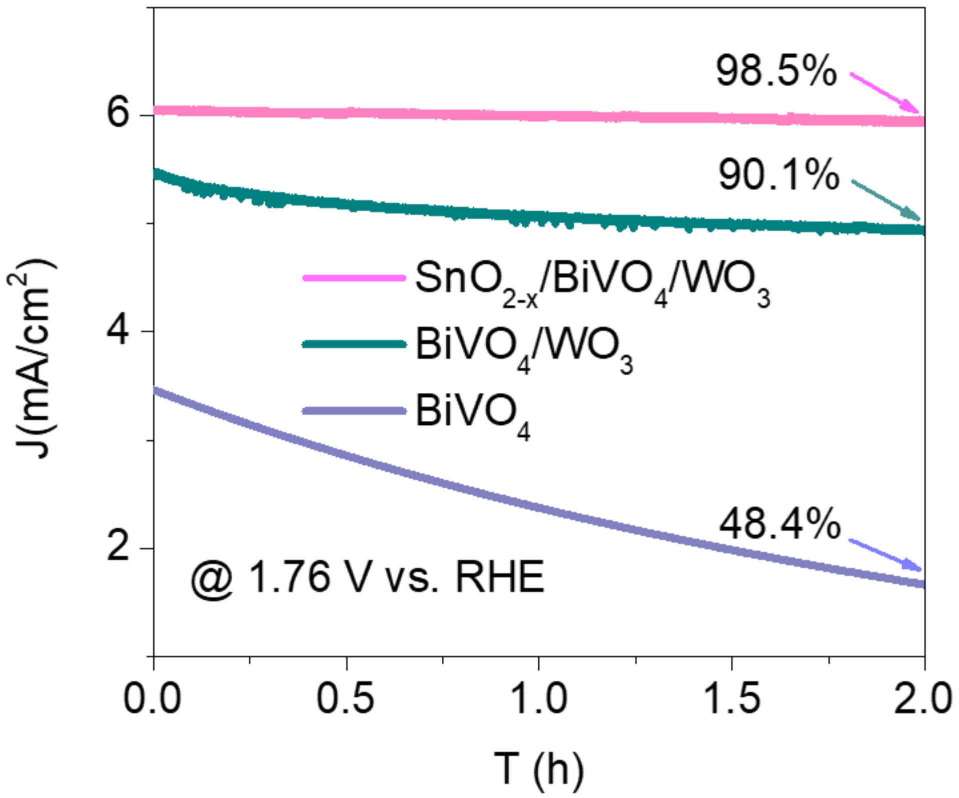

Supplementary Fig. 12. Potentiostatic i-t of $BiVO_4$, $BiVO_4/WO_3$ and $SnO_{2-x}/BiVO_4/WO_3$ photoanodes. Reaction conditions: 1.76 V vs. RHE under AM 1.5M illumination in 0.4 M $NaHCO_3$ electrolyte.

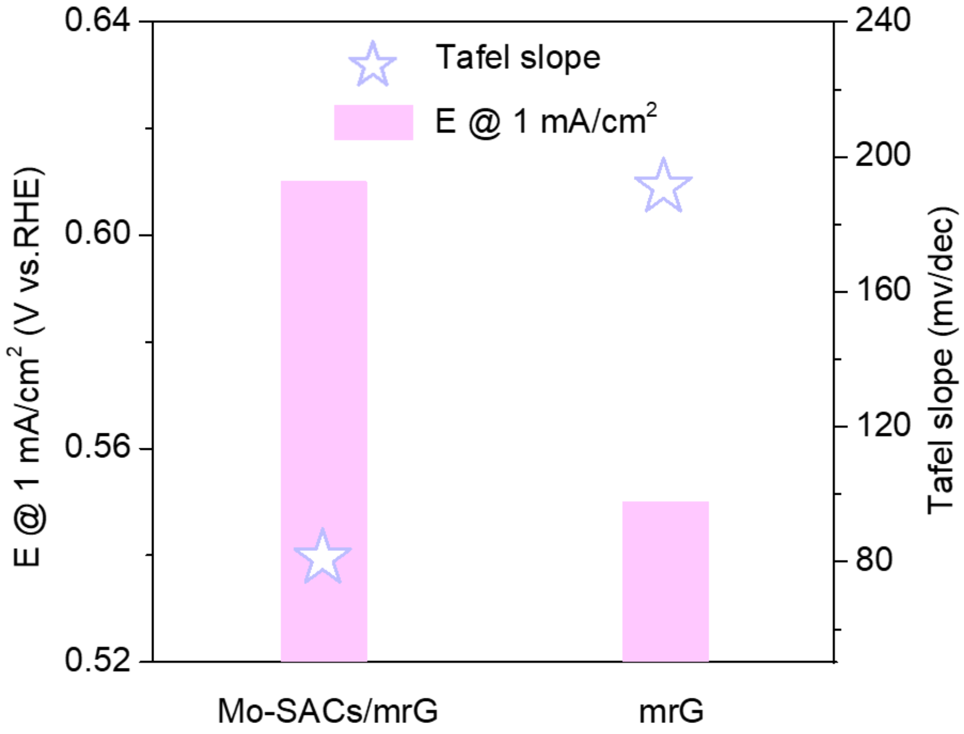

Supplementary Fig. 13. Electrochemical performance of Mo-SACs/mrG and mrG *via* RRDE. Onset potential and Tafel slope of Mo-SACs/mrG and mrG in O_2-purged 0.4 M $NaHCO_3$ electrolyte.

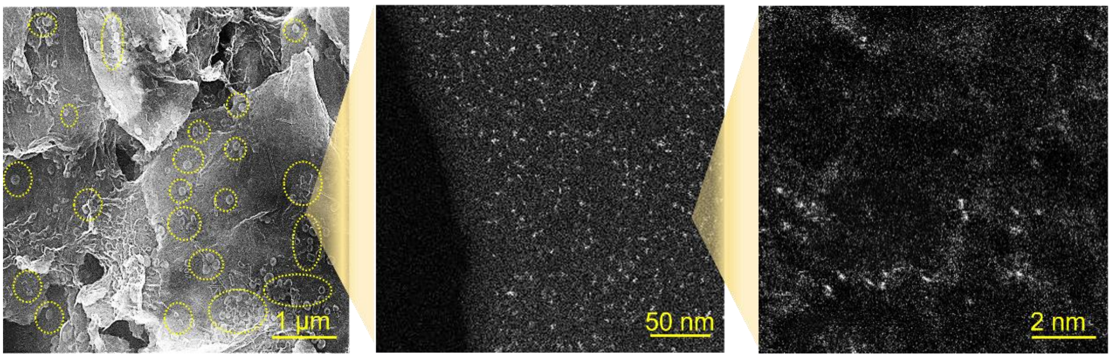

Supplementary Fig. 14. SEM and HAADF-STEM images of PTFE@Mo-SACs/mrG-GDE with higher magnification from left to right. PTFE particles decorated on the nanosheets are indicated by the yellow dashed circle. Scale bars from left to right: 1 μm, 50 nm, and 2 nm.

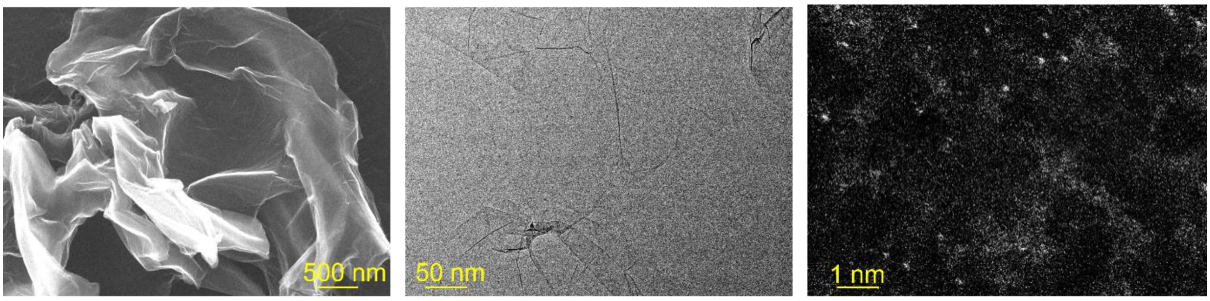

Supplementary Fig. 15. SEM, HR-TEM and HADDF-STEM images of Mo-SACs/mrG. The single metal atoms were the only metal species present.

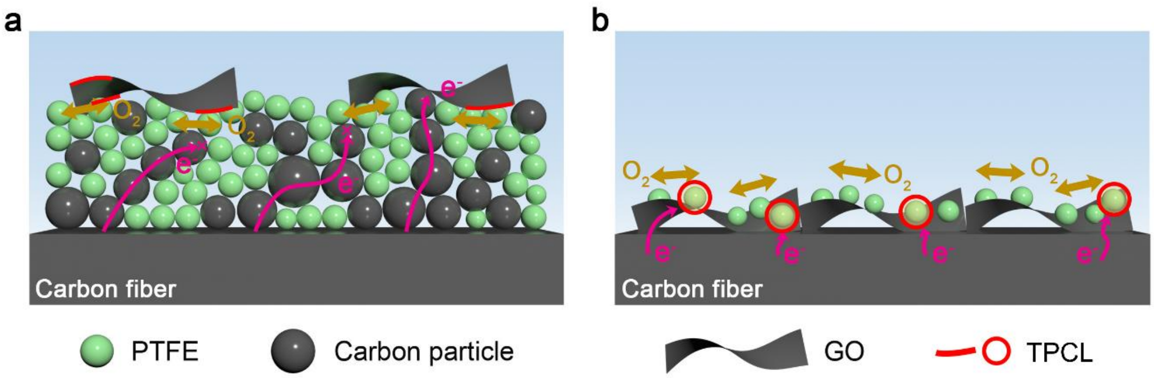

Supplementary Fig. 16. Schematic of the oxygen and electron transfer process. a, Mo-SACs/mrG-GDL and b, PTFE@Mo-SACs/mrG-GDE. Conventional GDL electrode demonstrated that a thick microporous gas diffusion layer composed of PTFE and carbon-black particles is introduced between the Mo-SACs/mrG nanosheets catalysts and carbon fiber (current collector) in order to enhance the oxygen diffusion efficiency to boost the three-phase contact line (TPCL). Even though, a thick and insulated GDL will slow electron transfer from carbon fiber cloth to Mo-SACs/mrG nanosheets. In contrast, PTFE@Mo-SACs/mrG-GDE in b showed that the quasi-nanoarray aerophilicity areas generated by the evenly distributed PTFE nanoparticles on the Mo-SACs/mrG nanosheets are able to simultaneously contribute to compatible three-phase contact line (TPCL) and significantly enhanced electron transfer compared to the conventional GDL electrode.

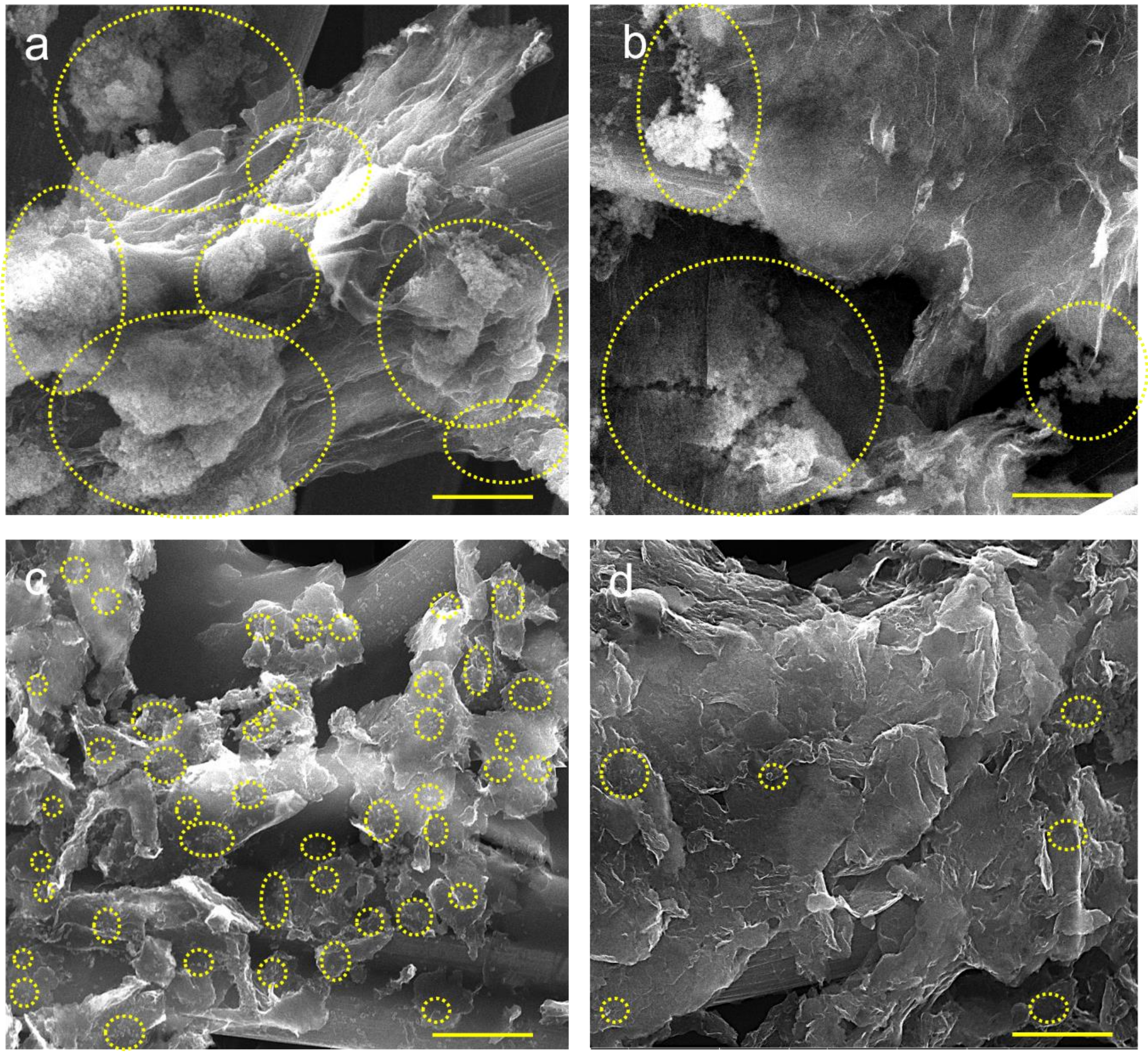

Supplementary Fig. 17. SEM of PTFE@Mo-SACs/mrG-GDE with different PTFE loading ratios. The loading ratios between PTFE and Mo-SACs/mrG from a to d were 20:1, 8:1, 2:1 and 0.2:1, respectively. Scale bar: 5 μm.

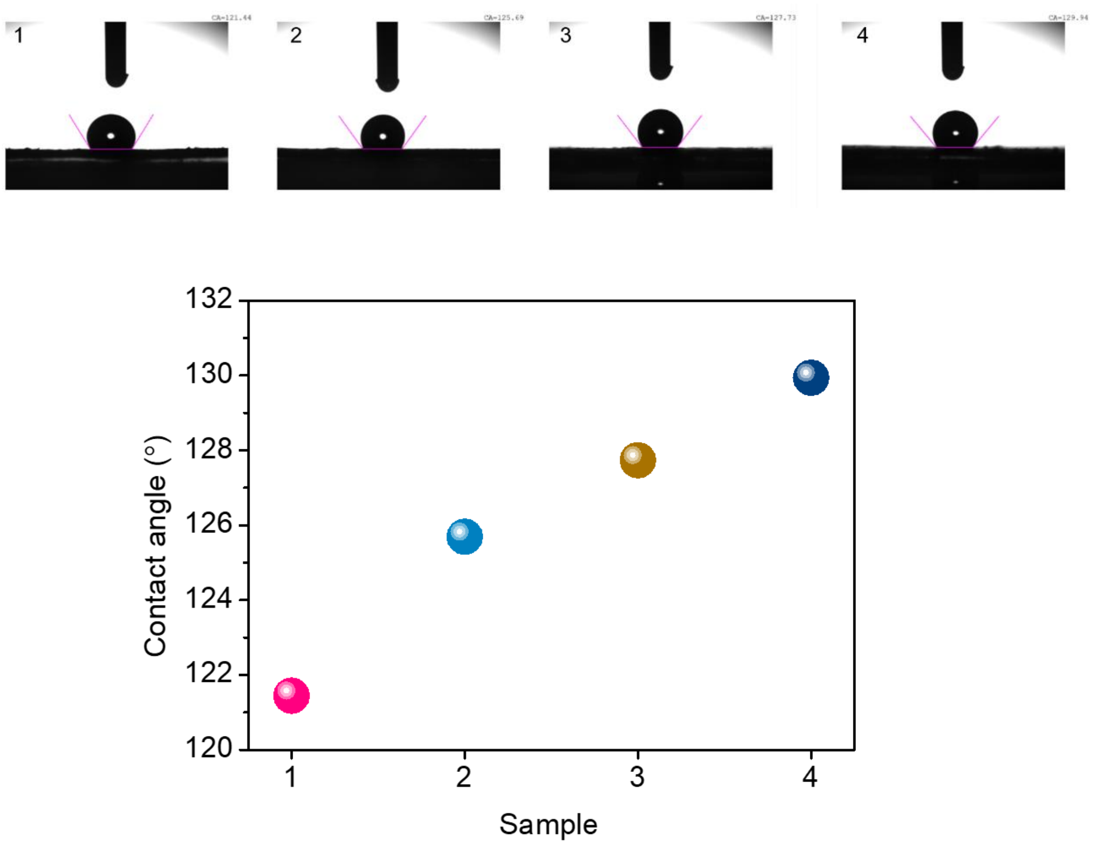

Supplementary Fig. 18. Contact angles of four kinds of PTFE@Mo-SACs/mrG-GDE with various mass ratios between PTFE and Mo-SACs/mrG. The mass ratios of PTFE and Mo-SACs/mrG of samples 1, 2, 3 and 4 were 0.2:1, 2:1, 8:1 and 20:1, respectively.

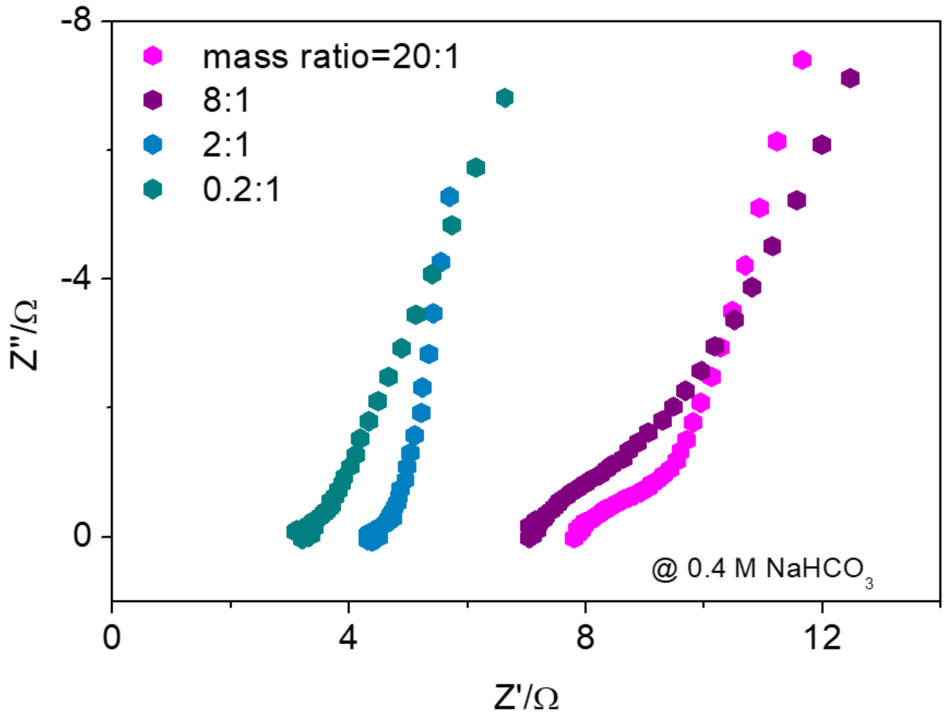

Supplementary Fig. 19. Electrochemical impedance spectra of four kinds of PTFE@Mo-SACs/mrG-GDE. The increasing loading mass ratio between PTFE and Mo-SACs/mrG leads to the increasing electrode resistance. The electrolyte was O_2-purged 0.4 M $NaHCO_3$.

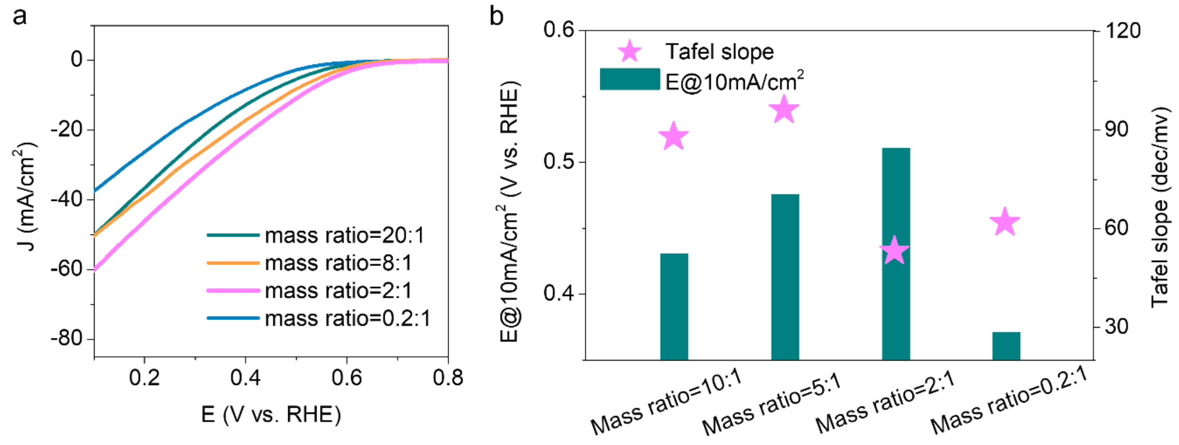

Supplementary Fig. 20. Electrochemical performance of four kinds of PTFE@Mo-SACs/mrG-GDE with various PTFE loading ratios. a, LSV scan of four kinds of PTFE@Mo-SACs/mrG-GDE with different PTFE loading ratios. b, Onset potential and Tafel slope of four kinds of PTFE@Mo-SACs/mrG-GDE with different PTFE loading ratios. Reaction is under O_2-purged 0.4 M $NaHCO_3$ electrolyte.

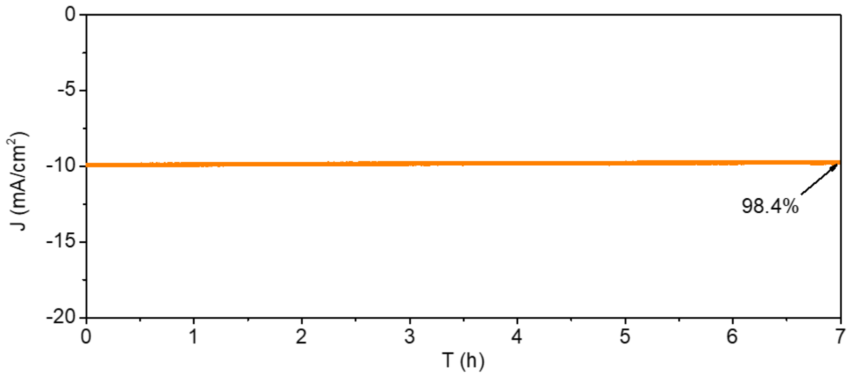

Supplementary Fig. 21. Potentiostatic i-t curve of PTFE@Mo-SACs/mrG-GDE at 0.5 V vs. RHE. Overall, 98.4% of the current density was maintained after 7 hours of testing in an 0.4 M $NaHCO_3$ electrolyte.

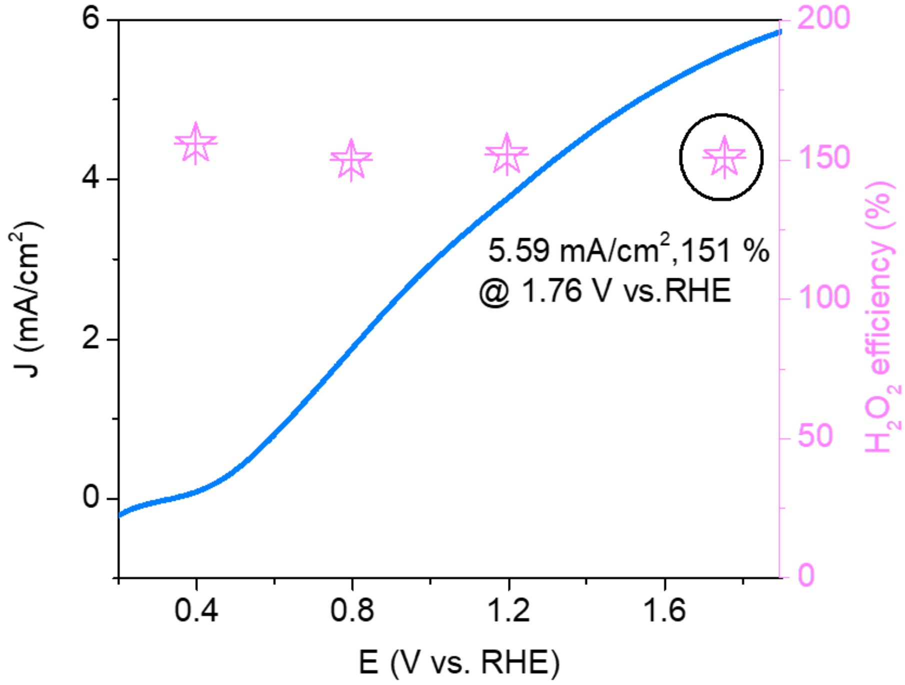

Supplementary Fig. 22. LSV scan of curve of SnO_{2-x}/$BiVO_4$/WO_3|| PTFE@Mo-SACs/mrG-GDE PEC cell under a three-electrode system and the H_2O_2 FE at different applied voltages. The current density at 1.76 V vs. RHE was 5.59 mA/cm^2 with an H_2O_2 FE of 151%.

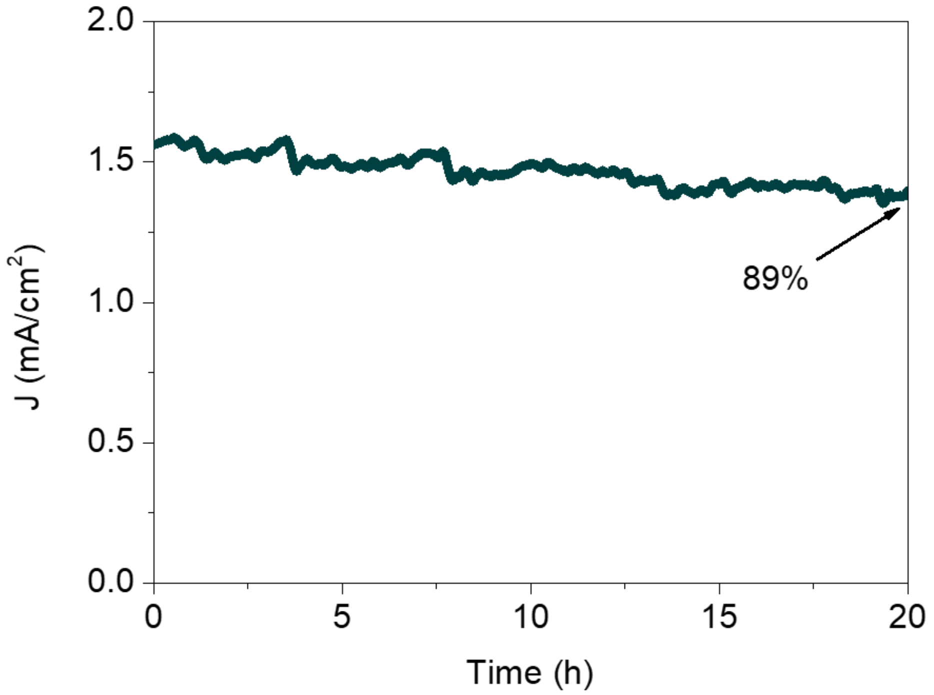

Supplementary Fig. 23. Potentiostatic i-t curve of a one-cell configuration PEC device under bias-free conditions. Overall, 89% of the current density was maintained after 20 hours of testing in an 0.4 M $NaHCO_3$ electrolyte.

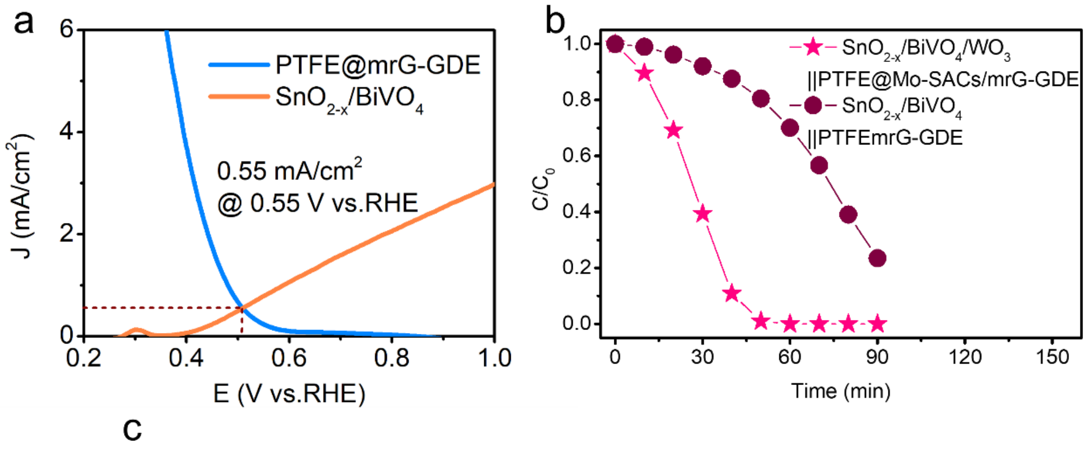

Supplementary Fig. 24. Control experiments using $SnO_{2-x}/BiVO_4$|| PTFE@mrG−GDE as the artificial leaf. a, Coupled LSV scan of the $SnO_{2-x}/BiVO_4$ photoanode and PTFE@mrG−GDE cathode. b, Degradation of 15 ppm NP of two kinds of artificial leaf (7 cm^2). Reaction media: 15 mL of 0.4 M $NaHCO_3$ solution with 0.04 ppm Mn(II).

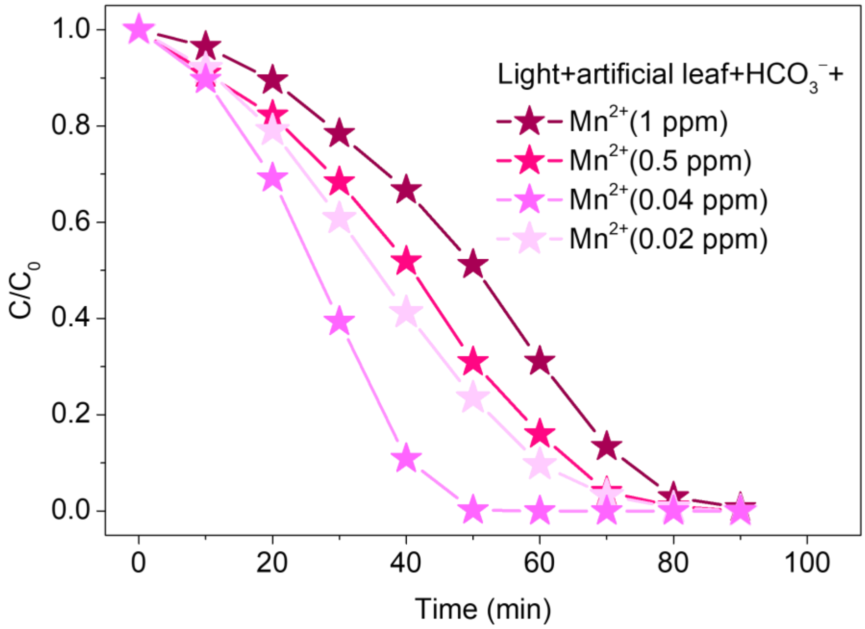

Supplementary Fig. 25. Degradation of 15 ppm 4-nitrophenol (NP) with different amounts of the Mn^{2+} catalyst. More than 99.5% of pollution was removed in 50 min, 80 min, 90 min and 90 min with Mn^{2+} concentrations of 0.02 ppm, 0.04 ppm, 0.5 ppm and 1 ppm, respectively. Reaction electrolyte: 15 mL of 0.4 M $NaHCO_3$ aqueous solution.

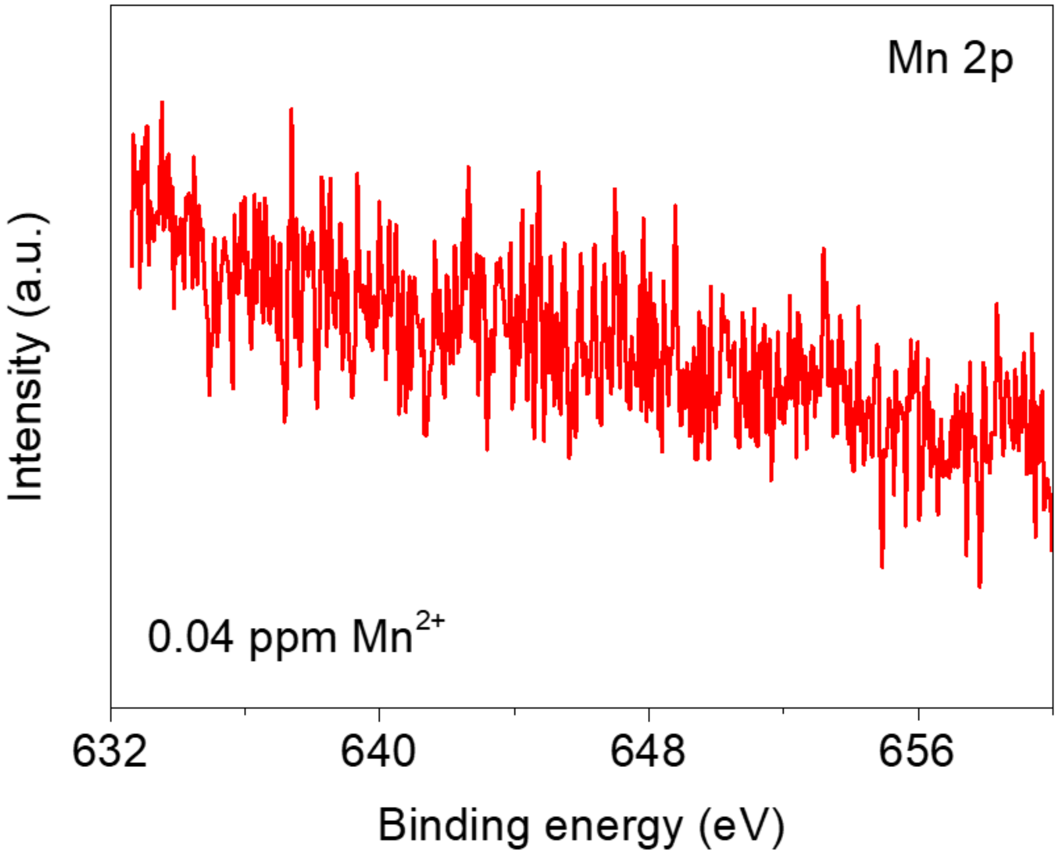

Supplementary Fig. 26. XPS spectrum of Mn on the $SnO_{2-x}/BiVO_4/WO_3$ photoanode after degradation tests. No signal was observed with 0.04 ppm Mn^{2+} catalyst. Reaction electrolyte: 15 mL of 0.4 M $NaHCO_3$ aqueous solution with 15 ppm 4-nitrophenol (NP).

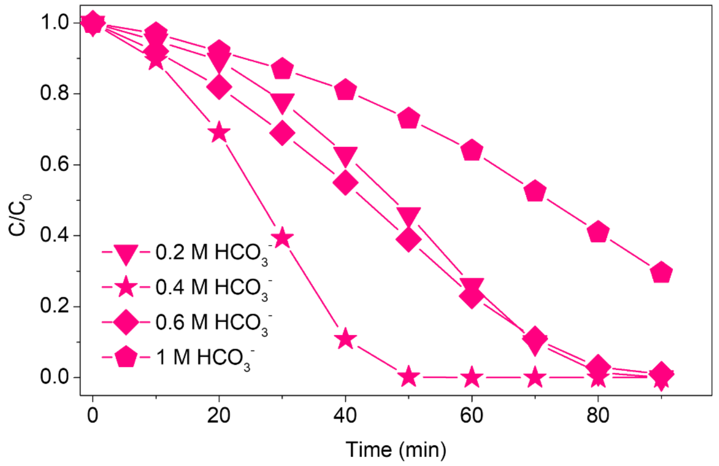

Supplementary Fig. 27. Degradation of 15 ppm NP with different bicarbonate concentrations. The reaction volume was 15 mL, and the Mn^{2+} catalyst amount was 0.04 ppm. Overall, 100%, 100 %, 99 % and 70% of pollution was removed in 90 min at $NaHCO_3$ concentrations of 0.2 M, 0.4 M, 0.6M and 1 M, respectively.

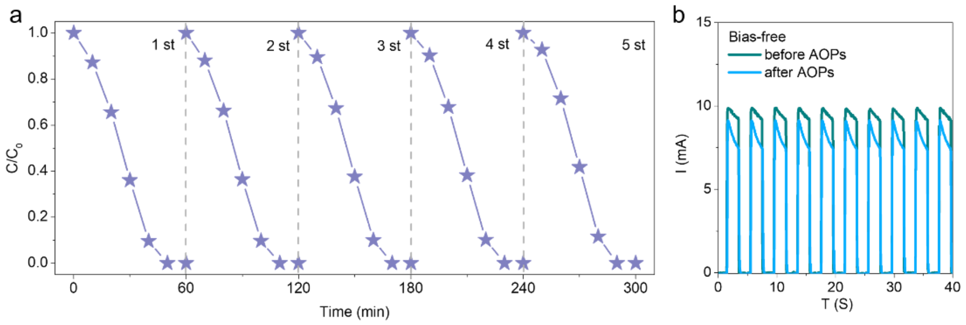

Supplementary Fig. 28. Durability test of degradation. a, Repeated degradation tests and b, the photocurrent–time profiles of $SnO_{2-x}/BiVO_4/WO_3$|| PTFE@Mo-SACs/mrG-GDE before and after degradation.

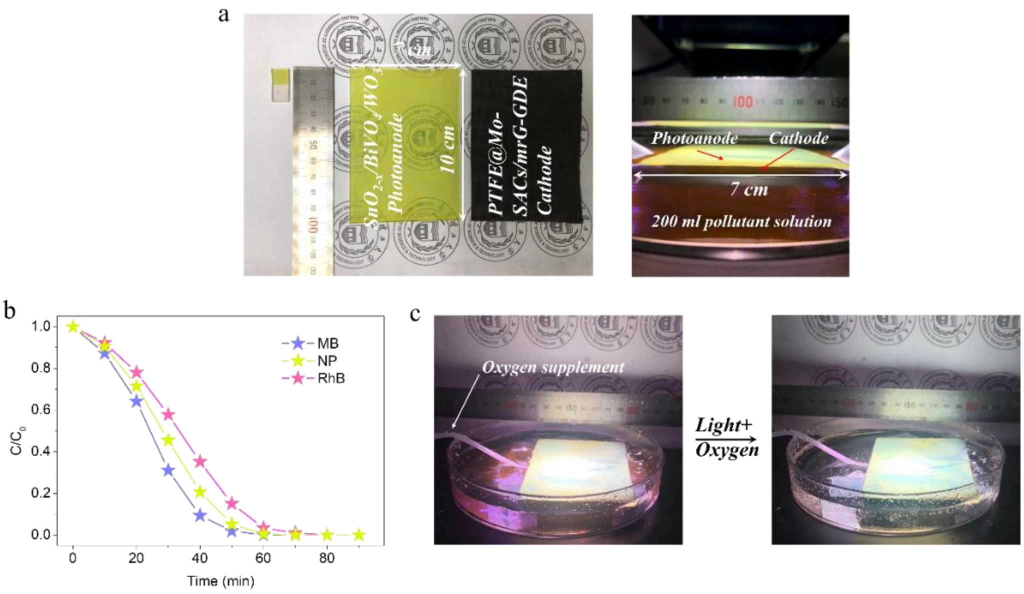

Supplementary Fig. 29. Degradation of 200 mL synthetic wastewater with 70 cm^2 artificial leaf. a, Photographs of the 7 × 10 cm^2 $SnO_{2-x}/BiVO_4/WO_3$ photoanode and PTFE@Mo-SACs/mrG-GDE cathode and large area (~70 cm^2) artificial leaf in 200 mL synthetic wastewater. b, Degradation of synthetic wastewater containing a mixture of 5 ppm rhodamine B, 5 ppm methylene blue and 5 ppm NP with input of only oxygen and sunlight. c, Optical image of the degradation process enabled by the artificial leaf with input of only solar light and oxygen.

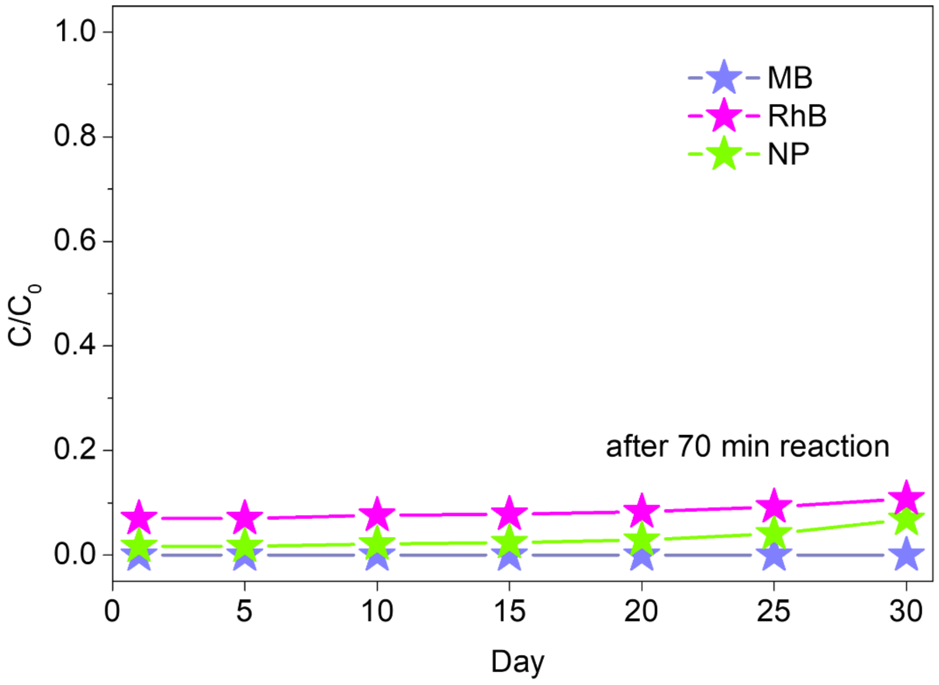

Supplementary Fig. 30. Stability measurement of the 70 cm^2 artificial leaf in one month. Pollutant remaining after 70 minutes degradation tests every 5 days. Wastewater sample: 200 mL synthetic solution containing 5 ppm rhodamine B, 5 ppm methylene blue and 5 ppm NP.

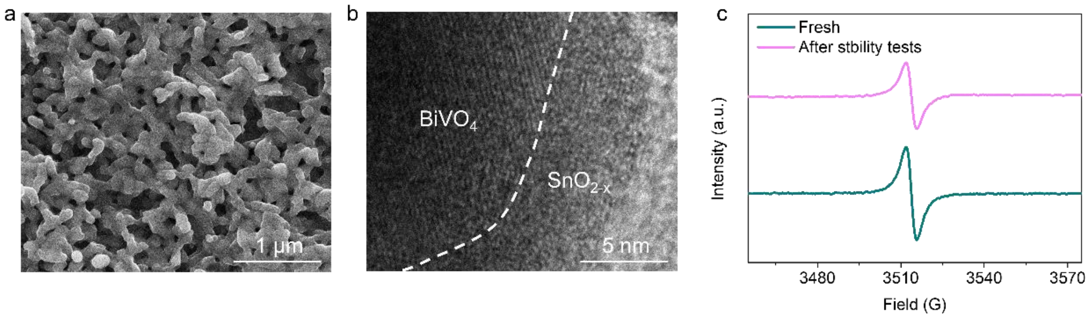

Supplementary Fig. 31. (a) SEM of $SnO_{2-x}/BiVO_4/WO_3$ photoanode after long-term stability tests. (b) HR-TEM of $SnO_{2-x}/BiVO_4/WO_3$ photoanode after long-term stability tests. (c) EPR spectra of $SnO_{2-x}/BiVO_4/WO_3$ photoanode before and after long-term stability tests.

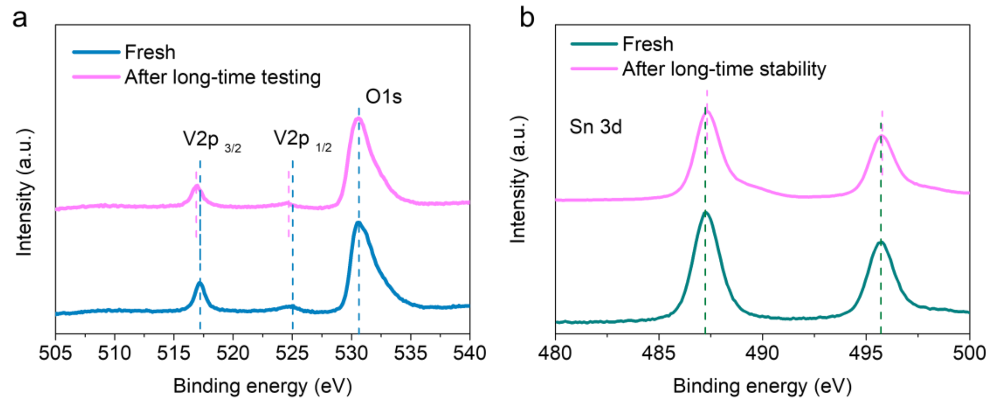

Supplementary Fig. 32. Comparisons of XPS results of $SnO_{2-x}/BiVO_4/WO_3$ before and after long-term testing. (a) V 2p and O1s and (b) Sn 3d.

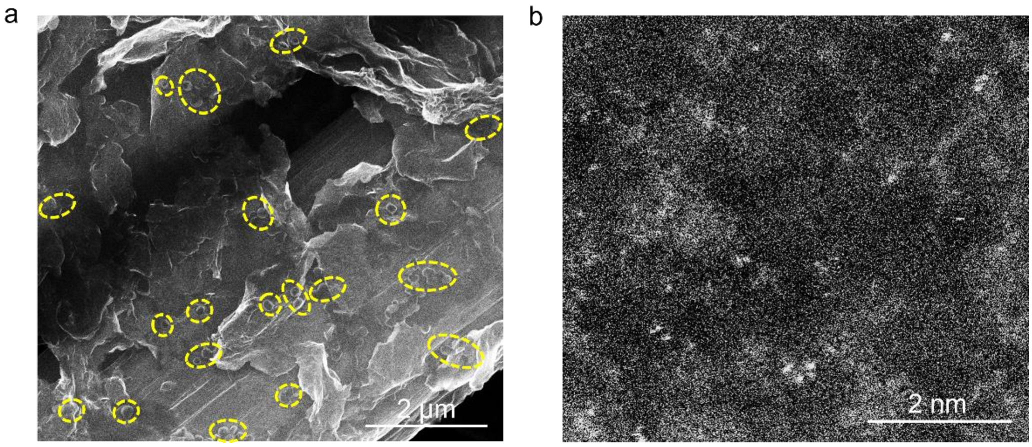

Supplementary Fig. 33. (a) SEM of Mo-SACs/mrG-GDE cathode after long-term stability tests. (b) HAADF-STEM of $SnO_{2-x}/BiVO_4/WO_3$ after long-term stability tests.

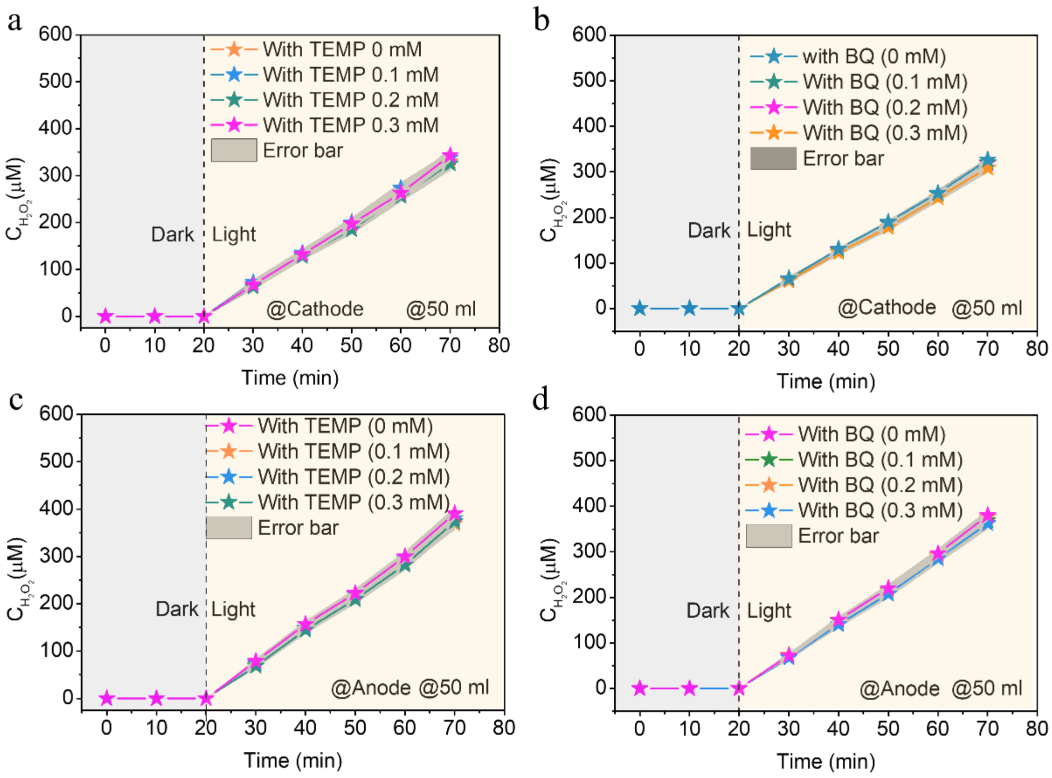

Supplementary Fig. 34. The effect of BQ and TEMP on the H_2O_2 production over cathode (a,b) and anode (c,d). Reaction conditions: 0.4 M $NaHCO_3$ electrolyte (50 ml for each cathode and anode in H-cell), BQ (0.10, 0.20 and 0.30 mM), TMPA (0.10, 0.20 and 0.30 mM), O_2-sturated and bubbled all the time, AM 1.5 illumination.

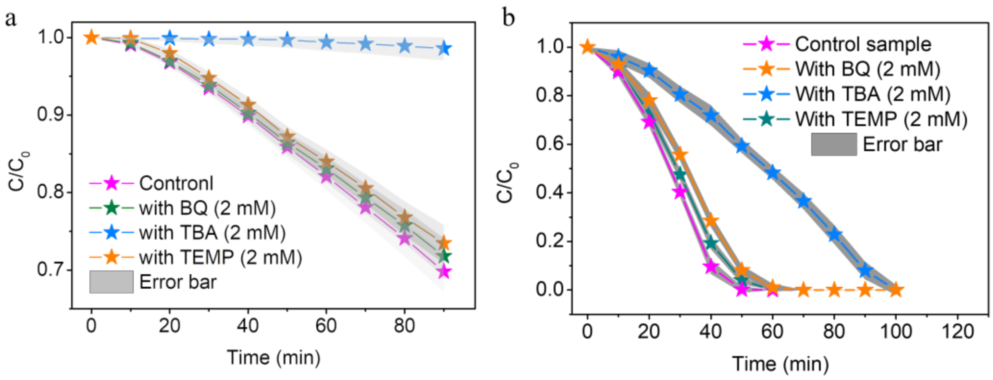

Supplementary Fig. 35. Degradation effect under different radical scavengers on 4-nitrophenol (4-NP) in the self-cycled photo Fenton-like system. Reaction conditions: 0.4 M $NaHCO_3$ electrolyte (15 ml) with 10 ppm 4-NP, concentration of Mn^{2+} is 0.4 ppm, O_2-sturated and bubbled all the time, AM 1.5 illumination.

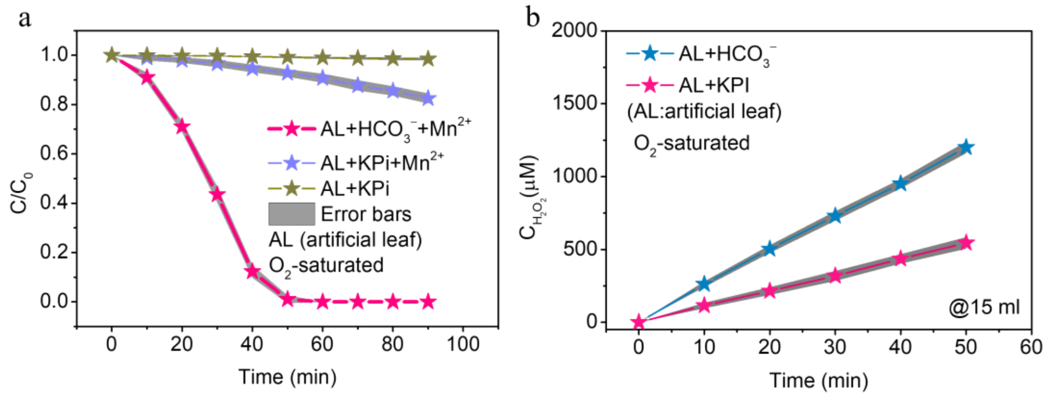

Supplementary Fig. 36. (a) The degradation effect in KPi electrolyte in the reaction system. (b) The H_2O_2 concentration in the self-cycled Fenton-like reaction system with different electrolyte. Reaction conditions: (a) 0.4 M $NaHCO_3$ or 0.4 M KPi electrolyte with the same pH value (15 ml) with 10 ppm 4-NP, concentration of Mn^{2+} is 0.4 ppm, O_2-sturated and bubbled all the time, AM 1.5 illumination in the degradation tests. (b) 0.4 M $NaHCO_3$ or 0.4 M KPi electrolyte with the same pH value (15 ml), O_2-sturated and bubbled all the time, AM 1.5 illumination in the degradation tests in the H_2O_2 concentration measurement.

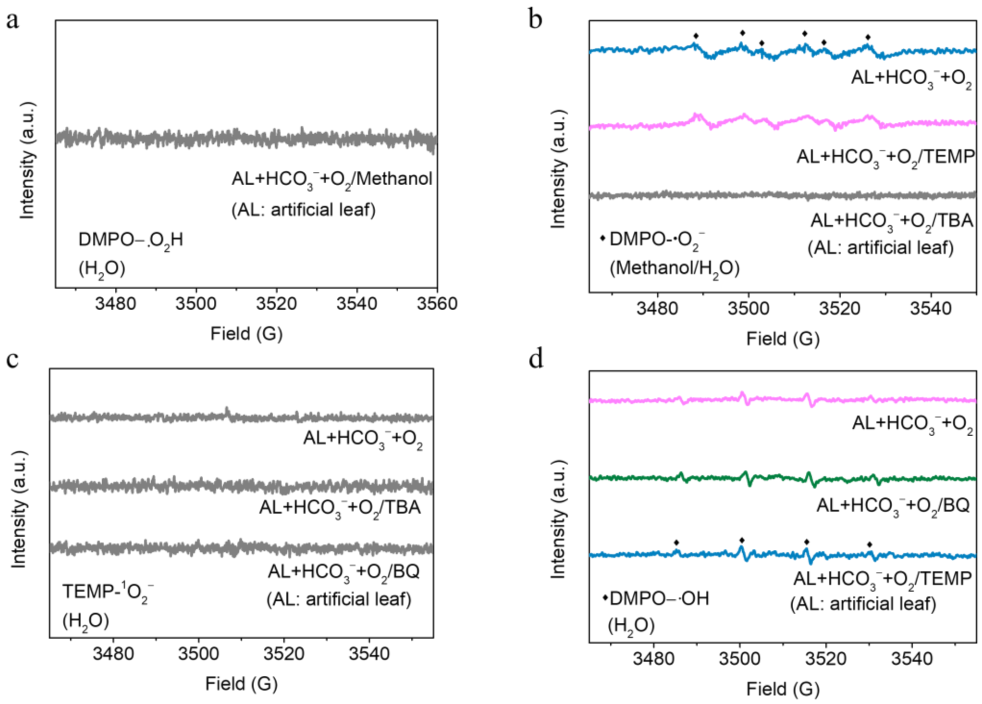

Supplementary Fig. 37. EPR response of (a) $\cdot O_2H$, (b) $\cdot O_2^-$, (c) $^1O_2$ and (d) $\cdot OH$ in the self-cycled photo-Fenton-like system (without Mn^{2+}) companied with different radical scavenger. Reaction conditions: 0.4 M $NaHCO_3$ electrolyte (15 ml), O_2-sturated and bubbled all the time, AM 1.5 illumination for 15 min.

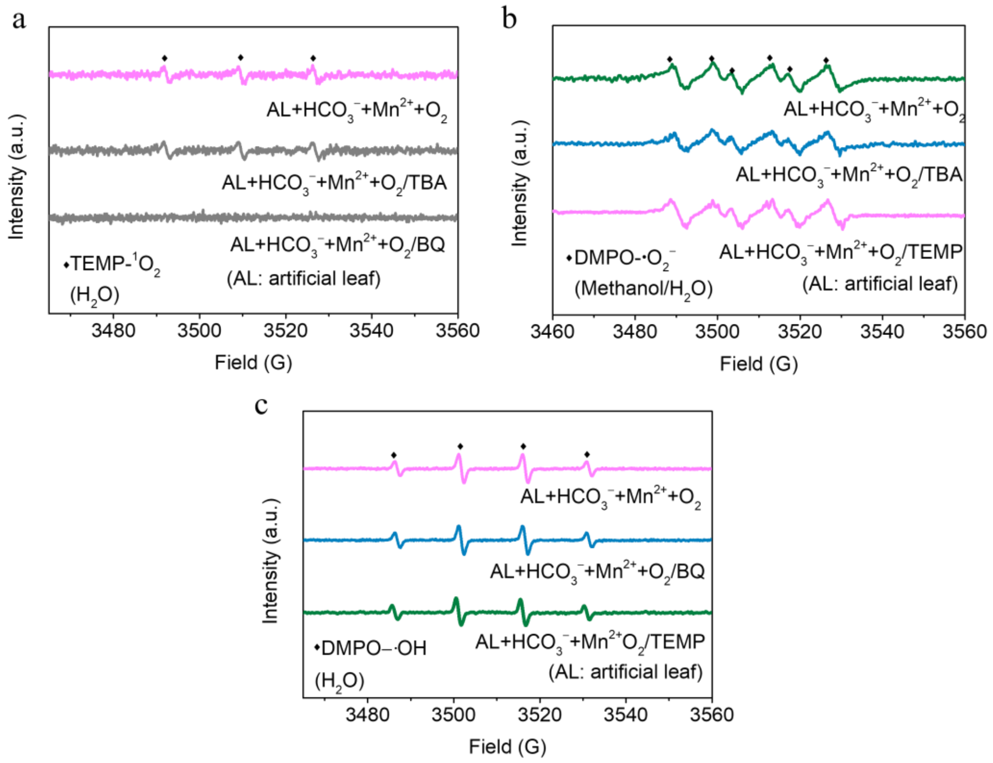

Supplementary Fig. 38. EPR response of (a) $^1O_2$, (b) $\cdot O_2^-$, (c) $\cdot$OH in the self-cycled photo-Fenton-like system (with Mn^{2+}) companied with different radical scavenger. Reaction conditions: 0.4 M $NaHCO_3$ electrolyte (15 ml), concentration of Mn^{2+} is 0.4 ppm, O_2-sturated and bubbled all the time, AM 1.5 illumination for 15 min.

Electrode Component	H_2O_2 production rate (μmol*cm^{-2}*min^{-1}) With bias (bias-free)	STH efficiency (%) With bias (bias -free)	With/without PV device	Two-electrode/three-electrode	Applied Bias (V vs. RHE)	Year	Reference
$SnO_{2-x}/BiVO_4/WO_3$‖Mo-SACs/mrG	2.63(1.76)	2.18(1.46)	no	both	1.76(Bias-free)	2022	This work
$SnO_{2-x}/BiVO_4$	0.99	1.87	no	Three-electrode	1.23	2020	23
RuO_x/TNR‖AQ/Graphite	(0.35)	(0.66)	no	Two-electrode	Bias-free	2021	39
P-Mo-$BiVO_4$‖AQ-CNT/C	0.66(0.16)	1.25(0.30)	no	both	1 (Bias-free)	2020	40
AQ-DSPECs	0.5	0.95	no	Three-electrode	0.13	2020	41
$NiFeO_x$-$BiVO_4$‖PTTh	0.4	0.75	no	Three-electrode	0.65	2020	42
$WO_3/BiVO_4$	0.084	0.16	no	Three-electrode	0.56	2016	43
m-WO_3‖carbon paper-$Co^{II/III}$(Ch)	(0.2)	(0.38)	no	Two-electrode	Bias-free	2016	44
$Al_2O_3/WO_3/BiVO_4$	1.0	1.89	no	Three-electrode	1.5	2017	45
$BiVO_4$‖carbon	(0.48)	(0.91)	no	Two-electrode	Bias-free	2018	46
$WO_3/BiVO_4$‖carbon	(0.13)	(0.25)	no	Two-electrode	Bias-free	2017	47
FeOOH-$BiVO_4$‖carbon paper-$Co^{II/III}$(Ch)	(0.2)	(0.38)	no	Two-electrode	Bias-free	2017	48
PSK/O-BP‖α-$NiFeO_x$/CP	(1.74)	(1.41)	($MAPbI_3$) perovskite solar cell	Two-electrode	Bias-free	2021	49

Supplementary Table 1. Comparison table of with the state-of-the-art PEC systems toward H_2O_2 production

Supplementary discussion

1. **The solar utilization efficiency**. One of the significant goals of our system is to build a sustainable AOPs system with self-generated H_2O_2 based on the PEC technology. A higher H_2O_2 generation rate is highly expected to accelerate the rate of AOPs due to the rapid consumption of H_2O_2. Based on that concept, kinetic optimization of photoanode and cathode is carefully and deliberately investigated to raise the H_2O_2 generation rate, namely, solar-to-H_2O_2 (STH) efficiency, as much as possible. Even though, we expect that the STH efficiency can be substantially raised by introducing the state-of-art photovoltaic (PV) in the PEC system because the only part range of solar energy is absorbed (<510 nm) due to the relatively large band gap of the $SnO_{2-x}/BiVO_4/WO_3$ photoanode (~2.45 eV). Therefore, the combination of a PV cell with a complementary absorption range (e.g. ~1.2 eV) could fulfill a much broader absorption range with the extra driving force, which could lead to a higher working current density of the tandem PEC system and the artificial leaf. According to previous theoretical research[1,2], the unassisted working current density is expected to be raised by 3-4 times when a qualified perovskite cell was combined.

2. **Supplement of oxygen**. In this work, the oxygen supplement for the 2e-ORR in the cathode relies on the aeration from a high-pressure oxygen bottle during the whole reaction process. Therefore, it would make large progress if the oxygen/air could naturally diffuse into the cathode without external compression. Recently, a few researchers reported the concept of a deliberately designed ORR cathode with a superhydrophobic interface that allows the rapid and natural transport of O_2 from the

atmosphere to the cathode through the backside of the electrode[3,4]. Our work, on the other hand, takes care of both conductivity and oxygen diffusion efficiency by creating quasi-nanoarray aerophilicity areas only on the electrocatalysts coating side (front side). Therefore, it is reasonable to believe that an “asymmetric sandwich” electrode with modified electrocatalysts coating the front side and oxygen natural diffusion backside could avoid aeration energy consumption with improved electrocatalysis performance at the same time, which is believed to substantially enhance the feasibility of our device application.

3. **Electrolyte in the natural environment**. The environmental benignity is one of the most prospecting merits of our device because the reactors and the products involved are only oxygen, water and hydrogen peroxide and the electrolyte is bicarbonate aqueous solution, which is ubiquitous in groundwater, showing strong competitiveness sustainably compared to the traditional AOPs. However, the relatively high electrolyte concentration (0.4 M HCO_3^-) is necessary for high H_2O_2 generation current density in the PEC system. Therefore, we expect a potential electrolyte with a lower concentration for high H_2O_2 generation current density in further research.

We hope that this discussion of the limitation and potential approaches will inspire future research toward further exploration of effective and sustainable wastewater treatment as well as efficient solar-fuel production/utilization devices.

Supplementary References

1. Beranek R. Selectivity of Chemical Conversions: Do Light-Driven Photoelectrocatalytic Processes Hold Special Promise? *Angew. Chem. Int. Ed.* **58**, 16724-16729 (2019).
2. Hu S. Membrane-less photoelectrochemical devices for H2O2 production: efficiency limit and operational constraint. *Sustain. Energy Fuels* **3**, 101-114 (2019).
3. Xu J*, et al.* Organic wastewater treatment by a single-atom catalyst and electrolytically produced H2O2. *Nat. Sustain* **4**, 233-241 (2020).
4. Yu J, Li BQ, Zhao CX, Liu JN, Zhang Q. Asymmetric Air Cathode Design for Enhanced Interfacial Electrocatalytic Reactions in High-Performance Zinc-Air Batteries. *Adv. Mater*. **32**, e1908488 (2020).
