# Supplementary material for: Self-cycled photo-Fenton-like system based on an artificial leaf with a solar-to-H2O2 conversion efficiency of 1.46%
Source: Nat Commun. 2022 Aug 25;13:4982. doi: 10.1038/s41467-022-32410-0 (PMC9411154; doi:10.1038/s41467-022-32410-0)
Supplement: Supplementary file 3 — Description to Additional Supplementary Information [file 41467_2022_32410_MOESM3_ESM.pdf]

Description of Additional Supplementary files

File Name: Supplementary Movie 1

The video shows an organic dye mixture containing 5 ppm rhodamine B (Rh. B), 5 ppm methylene blue (MB) and 5 ppm NP being degraded over the 7×10 cm^2 artificial leaf under light illumination.
